# Hospital utilization and telemedicine preferences in patients with late-stage Parkinson’s disease and caregivers

**DOI:** 10.1016/j.prdoa.2024.100296

**Published:** 2024-12-31

**Authors:** Seo Hyun Hong, Seoyeon Kim, Seungmin Lee, Bora Jin, Jung Hwan Shin, Kyung Ah Woo, Han-Joon Kim

**Affiliations:** a Seoul National University College of Medicine, Seoul, Korea; b Department of Neurology and Movement Disorders Center, Seoul National University Hospital, College of Medicine, Seoul National University, Seoul, Korea

**Keywords:** Parkinson’s disease, Late-stage, Hospital utilization, Telemedicine, Rehabilitation

## Abstract

**Background:**

There remains a significant gap in systematic research on healthcare utilization behaviors and the influencing factors for patients with Parkinson’s disease (PD), particularly those in late stages.

**Methods:**

PD patients in late stage (Hoehn and Yahr (HY) stages 4 and 5) and their caregivers from Seoul National University Hospital Movement Disorders Clinic participated. A total of 103 respondents completed a questionnaire covering medical utilization behaviors, perceptions of face-to-face and telemedicine consultations, and additional medical service needs. Descriptive analysis was conducted based on HY stage, age, and travel time to the hospital.

**Results:**

82.1% of patients in HY4 make more than 50% of in-person visits by themselves or with caregivers, compared with only 38.9% of patients in HY5. Despite proxy visits by caregivers were common, audiovisual or written materials about the patient’s condition were underused (63% answered ‘never’). Over three-quarters of patients did not receive rehabilitation therapy, mainly due to mobility issues and the lack of nearby facilities. One third of respondents were open to telemedicine, with differing preferences between age groups. 22% of HY5 patients or their caregivers were willing to pay more for telemedicine than in-person visit.

**Conclusion:**

This study seeks to understand hospital use patterns and needs in late-stage PD patients and their caregivers. Current treatment framework for PD has areas that, if improved, could significantly enhance the quality of care. Telemedicine offers an opportunity to enhance PD education and assessment, introducing new methods for remotely measuring symptoms.

## Highlights

• Telemedicine holds potential for addressing the limitations associated with proxy visit and enhancing the accuracy of patient evaluation.
• For certain patients or caregivers, telemedicine is regarded not merely as an alternative healthcare service.
• Activating education through telemedicine and video calls or social media is needed.
• Institutional support is needed for effective enhancement of current medical system for PD.

## 1 Background

Parkinson's disease (PD) is a chronic progressive neurological disorder characterized by dopaminergic cell loss in the substantia nigra [1], [2]. As PD advances, patients exhibit levodopa-unresponsive motor symptoms and develop non-motor symptoms, leading to dependence on assistive devices or caregivers. The patient in this stage is characterized by substantial disability, motor complications that compromise the ability to perform basic daily activities, profound dysphagia, frequent falls, and dementia, all necessitating external support for daily functions [3]. This stage is commonly referred to as the late stage or the advanced stage, yet the definition of the term lacks a universally accepted consensus [4]. Late-stage PD patients, who rely on assistive devices or caregiver support, often experience discomfort when visiting hospitals, resulting in hospital utilization patterns that differ from those of patients in earlier stages. Consequently, their perspectives on current and future medical services, including an increased interest in telemedicine, may also differ. A study surveying 554 PD patients across South Korea found that nearly 70% favored telemedicine services, though it did not exclusively focus on late-stage PD patients [5]. Despite this, in Korea, there remains a significant gap in systematic research on healthcare utilization behaviors and the influencing factors for PD patients, particularly those in late stages. Moreover, there is a scant comprehensive examination of the medical and caregiving burdens faced by late-stage PD patients and their caregivers within the existing framework of medical care, alongside their unmet needs.

We aimed to explore the hospital utilization behaviors and telemedicine perceptions of late-stage PD patients and their caregivers. Through this approach, we aim to enhance healthcare accessibility for PD patients and identify more effective methods for delivering medical services to this population.

## 2 Methods

We defined the patients with late-stage PD as the patients in Hoehn and Yahr stages 4 and 5 even in medication-on condition [6], [7]. Participants were enrolled consecutively from the outpatient clinic of Movement Disorders Clinic of Seoul National University Hospital. The questionnaire used in this study was elaborated by the movement disorders specialists and contained 17 multiple-choice items related to medical utilization behaviors of late-stage PD patients, perceptions of face-to-face and telemedicine consultations, and additional needs for medical services such as education and monitoring. ‘Telemedicine’ was defined as the delivery of any healthcare service at a distance, otherwise the patients can be provided only by in-person visit to the hospital. When the patient’s cognitive function was not impaired, the survey was answered primarily by the patient, sometimes after discussion with their caregiver if needed. When the patient has cognitive impairment, the survey was answered by the caregiver. The questionnaire was administered during the waiting time at clinic and took 15 min to complete.

The data obtained underwent descriptive analysis based on specified criteria including HY stage, age, and the travel time to the hospital. This study was performed in accordance with the Declaration of Helsinki. Informed consent was waived by the Seoul National University Hospital Institutional Review Board (SNUH IRB).

## 3 Results

The number of participants was 103, including late-stage PD patients and their caregivers. Among 103 participants, 67 patients were HY 4 and 36 were HY 5. (Table 1) About 69% reported that they visit the hospital every 3 to 6 months. 81.2 % in HY4 make more than 50% of in-person visits by themselves or with caregivers, compared with only 40% in HY5. Additionally, out of all patients, when travel time to the hospital was over 2 h, about 36% of visits were proxy visits by caregivers.

**Table 1:** Distribution of Respondents.

Age	Male	Female
40–49	0	1
50–59	2	0
60–69	9	3
70–79	28	15
80 ∼	29	15
**Severity**		
HY4	44	23
HY5	24	12
Total	68	35

Detailed responses from the respondents were provided in Table 2, Table 3. About 88% of respondents reported that the patient's condition was ‘sufficiently’ or 'mostly' conveyed through the caregiver. But only a quarter of caregivers provided audiovisual recordings or written memo describing the patient’s condition during proxy visits. Despite the prevailing view, some respondents (n=7) reported that the patient's condition was conveyed ‘insufficiently’.

**Table 2:** Response to the Survey.

		**HY4**	**HY5**
**Q. What is the typical interval of hospital visits for PD treatment?**	Shorter than a month	0	0
	1–3 months	12	9
	3–6 months	46	25
	6–9 months	7	2
	9–12 months	0	0
	Longer than 12 months	2	0
**Q. How frequently does the caregiver attend PD appointments without the patient?**	The patient always visits in person	41	10
	The patient attends more than 50 % of all medical appointments	11	4
	The patient attends less than 50 % of all medical appointments	5	2
	The caregiver always visits instead of the patient	7	19
**Q. When only caregivers visit, do you think the usual state of the patient at home is adequately communicated to the attending physician during outpatient treatment for PD?**	Always	11	15
	Often	20	13
	Sometimes	6	1
	Never	0	1
**Q. When only caregivers visit, do you show videos of the patients’ condition in the consultation room or communicate the patients’ condition to the attending physician via audio/video call?**	Always	2	4
	Often	2	0
	Sometimes	2	4
	Never	41	22
**Q. When only caregivers visit, do you provide the attending physicians with written notes, letters, or memos detailing the patient’s condition, written by either the patient or the caregiver?**	Always	6	6
	Often	4	2
	Sometimes	2	4
	Never	23	18
**Q. Are you receiving rehabilitation treatment for PD?**	Yes	18	8
	No	49	28
**Q. Have you ever received education or seen materials related to the treatment and management of PD?**	Yes	34	20
	No	33	16

**Table 3:** Perspectives on telemedicine.

		HY4	HY5
**Q. If telemedicine services were available for your condition, would you be willing to receive remote PD treatment and management via the internet instead of visiting the outpatient clinic in person?**	I am willing to receive telemedicine as the primary method of care, with in-person visits as needed	16	13
	I prefer in-person visits as the primary method of care, with telemedicine as an option when necessary	26	9
	I prefer in-person visits as the primary method of care, with telemedicine as an option when necessary	17	8
	Other comments	1	5
**Q. If telemedicine services were offered, would you be willing to use video calls on your phone for remote PD treatment instead of visiting the outpatient clinic in person?**	I am willing to receive telemedicine as the primary method of care, with in person visits as needed.	17	15
	I prefer in person visits as the primary method of care, with telemedicine as an option when necessary.	32	12
	I am not interested in receiving telemedicine services.	9	4
	Other comments	4	5
**Q. If telemedicine services were offered, would you be willing to use video calls on your phone for remote PD treatment instead of visiting the outpatient clinic in person?**	I am willing to receive telemedicine as the primary method of care, with in person visits as needed.	17	15
	I prefer in person visits as the primary method of care, with telemedicine as an option when necessary.	32	12
	I am not interested in receiving telemedicine services.	9	4
	Other comments	4	5
**Q. Would you be interested in using a remote monitoring system that can objectively record the patient’s condition, assess their usual state, and communicate the information to the primary care physician?**	Yes	45	27
	No	17	8
**Q. Would you be interested in participating in a remote rehabilitation program that you can do from home using video calling features?**	Yes	42	25
	No	25	10
**Q. Would you be interested in participating in a remote education program that you can do from home using video calling features?**	Yes	43	24
	No	24	9
**Q. If telemedicine services were offered, would you be willing to use video calls on your phone for remote PD treatment instead of visiting the outpatient clinic in person?**	I think the cost should be the same as in-person visits	24	12
	I think the cost should be lower than in-person visits	30	11
	I am willing to pay more than the cost of in-person visits if necessary.	5	6

Regardless of severity of the condition, more than three-quarters of patients do not receive rehabilitation therapy. 'Difficulty in mobility' was the most common reason (n=33), followed by 'lack of facilities/personnel offering rehabilitation therapy nearby.' (n=24) Regarding patient education, 52% stated that they had undergone some form of education on PD.

The proportion of respondents preferring telemedicine as their primary consultation method, with in-person visits as needed, was 31% for internet and 32% for video calls. Only 9% did not want to use telemedicine at all for both internet and video calls, indicating a minority compared to those who preferred using telemedicine either primarily or supplementarily.

A clear difference in preference for telemedicine services was observed across age groups. When comparing preferences for remote healthcare programs, 67% of patients aged 70 and above or their caregivers preferred 'remote rehabilitation programs' compared to 42% in under 70. Conversely, 'remote education programs' were more favored by the younger patient group than older group.

Opinions on telemedicine fees varied greatly with the disease's severity and the patient's age. Notably, 11 out of 88 respondents expressed their willingness to pay more than the current fees for in-person consultations. When examining the data by age group, only older patients (70 and above) and their caregivers were willing to pay more than the current fees for face-to-face consultations.

## 4 Discussion

As PD progresses, more and more proxy visits become inevitable in many patients due to mobility challenges. This likely leads to insufficient communication about patient’s symptoms from caregivers to doctors, which can be further compounded by the underutilization of written memos or audiovisual recordings on the patients. One research showed that caregivers often perceive the severity of a PD patient's disability and quality of life more negatively than the patients themselves, highlighting the potential for biased reporting [8]. To mitigate this, strategies promoting the use of written memos or audiovisual recordings are needed for accurately conveying the patient's condition remotely. Additionally, integrating telemedicine, such as video calls, with traditional consultations could allow physicians to directly observe and assess the patient's condition, thereby potentially enhancing the accuracy of diagnosis and treatment.

Patients in HY 5 or their caregivers showed a higher intention towards receiving remote consultations through the internet and video calls compared to those in HY 4. These results are in line with a previous study involving 86 patients with PD which compared patient satisfaction after experiencing both in-person and remote consultations [9]. Overall, no significant difference in satisfaction levels was observed between patients using in-person and telemedicine services. Particularly, higher satisfaction with telemedicine was noted among patients with more severe conditions, suggesting that telemedicine could be a valuable alternative that meets the needs of patients with severe symptoms. In another study, telemedicine has been found to provide high levels of satisfaction among patients with PD, especially those receiving speech-language or mental disorder treatments [10], [11]. Speech-language disorders and mental disorders are common in advanced PD and can be factors that reduce the effectiveness of in-person treatment. These findings suggest that telemedicine could be a useful alternative for PD patients with these conditions. Accessibility to and availability of in-person care is another important factor determining the preference to telemedicine. Due to the distance between the patients' home and their hospital, most patients in Korea cannot receive rehabilitation services at their hospital and they should find a rehabilitation clinic near their home. However, most of them have difficulty in finding a good one nearby and this is the reason why more than three-quarters of patients do not receive rehabilitation therapy. Therefore, many patients and caregivers want rehabilitation telemedicine.

Telemedicine may save both patients’ and caregivers’ time and money, but there is a trade-off as the quality of medical care may be lower than that of in-person visits [12]. According to a study, the video quality for telemedicine recordings was insufficient to accurately score all components of the motor UPDRS [13]. Consequently, opinions on what constitutes a fair price may vary among individuals. For these, we investigated patients' or their caregivers’ perceptions of appropriate fees for telemedicine services. A noteworthy finding is that some in the HY 5 group were willing to pay more than the current fees for in-person visits. This result highlights that late-stage PD patients or caregivers, especially those with severe conditions perceive that telemedicine has value that offset the high cost. This finding suggests that for certain patient groups, telemedicine is regarded not merely as an alternative healthcare service.

In the question about PD education, the experience of education on treatment and management of PD varied among participants depending on the age of the patient. Particularly, less than half of the 60–79 years old patients or their caregivers had received education on PD, whereas more than half of those aged 80 and above and their caregivers had. Regarding the sources of their education, participants mainly obtained information from pamphlets and leaflets provided by hospitals. In our medical system, due to the lack of time, patient education at clinic by treating physician is usually very quick and superficial. Instead, patients are usually provided with leaflets on the various aspects of PD and the link to the official patient education YouTube site of Korean Movement Disorders Society. Based on this, activating education through telemedicine and video calls or social media including YouTube could reduce spatial constraints and allow easier access to educational content, thereby improving the quality of PD management.

For the application of telemedicine on patients with PD, it is also necessary to assess the suitability of PD for remote consultations. Except for rigidity, which requires physical interaction between the examiner and the patient, primary motor symptoms such as bradykinesia and rest tremor can be assessed remotely, making telemedicine a feasible option. Furthermore, recent research using Wi-Fi routers installed in patients' homes expands the technical possibilities for telemedicine, offering new methods for remotely measuring patients' walking speed and movement. This device detects and analyzes radio waves that bounce off people, inferring their movements and walking speed. It also captures fluctuations in symptoms due to medication and their effect on daily functions [14], [15].

Our analysis clearly shows that the current treatment framework for PD has areas that, if improved, could significantly enhance the quality of patient care. Firstly, assuming the current in-person visit system will be maintained either entirely or partially, there should be a more proactive utilization of memos or audiovisual recordings. Secondly, telemedicine holds potential for addressing the limitations associated with proxy visit and enhancing the accuracy of patient evaluation. For HY 5 patients or caregivers, more respondents preferred to use telemedicine as their primary mode of care, compared to HY 4 who saw it more as a supplementary approach. To significantly improve the quality of life for patients with late-stage PD and their caregivers, the active implementation of telemedicine can be considered. Additionally, patient and caregiver education can be provided to PD patients through the internet or social media. Overall, these new methods can improve the management of patients, especially those in the late stage. To facilitate this advancement, institutional support is needed for effective enhancement of the current medical system for PD [16], [17], [18].

The limitation of this study includes a small sample size and the inability to differentiate between patient and caregiver responses. Additionally, the findings were limited to a single tertiary care center, which may not represent the entire PD patient and caregiver population. Furthermore, telemedicine is not allowed in Korea except in very limited circumstances, such as patients in prison or patients living in remote islands. None of video/voice calls using the patient's smartphone, remote monitoring or remote rehabilitation are allowed in Korea. Therefore, our questions in this study were 'if permitted, what is your opinion?' In Korea, the only use of video call/phone call at the clinic is when a caregiver visits without a patient. In that circumstance, the caregiver may call the patient using his/her phone and put the doctor on the phone. These circumstances should be considered when interpreting our results.

Future research should involve a larger, multi-site study that examines telemedicine preferences. Additionally, it would be valuable to explore the differences in telemedicine preferences and utilization between patients and their caregivers, as their needs and perspectives may vary.

## CRediT authorship contribution statement

**Seo Hyun Hong:** Writing – original draft, Formal analysis, Data curation. **Seoyeon Kim:** Writing – review & editing. **Seungmin Lee:** Writing – review & editing. **Bora Jin:** Writing – review & editing, Methodology. **Jung Hwan Shin:** Writing – review & editing, Methodology. **Kyung Ah Woo:** Writing – review & editing, Methodology. **Han-Joon Kim:** Writing – original draft, Supervision, Methodology, Funding acquisition, Data curation, Conceptualization.

## Funding

This research was supported by a grant of the Korea Health Technology R&D Project through the Korea Health Industry Development Institute (KHIDI), funded by the Ministry of Health & Welfare, Republic of Korea (grant number: HI23C0092).

## Declaration of competing interest

The authors declare that they have no known competing financial interests or personal relationships that could have appeared to influence the work reported in this paper.
